# Measuring the cost-effectiveness of a home-visiting intervention to promote early child development among rural families linked to the Rwandan social protection system

**DOI:** 10.1371/journal.pgph.0002473

**Published:** 2023-10-24

**Authors:** Chris Desmond, Kathryn G. Watt, Sarah K. G. Jensen, Erik Simmons, Shauna M. Murray, Jordan Farrar, Matias Placencio-Castro, Vincent Sezibera, Laura B. Rawlings, Briana Wilson, Theresa S. Betancourt

**Affiliations:** 1 Faculty of Health Sciences, SAMRC/Wits Centre for Health Economics and Decision Science, PRICELESS, University of Witwatersrand School of Public Health, Johannesburg, South Africa; 2 Centre for Rural Health, University of KwaZulu-Natal, Durban, KwaZulu-Natal, South Africa; 3 Boston College, School of Social Work, Chestnut Hill, Massachusetts, United States of America; 4 University of Massachusetts Boston, Boston, Massachusetts, United States of America; 5 Boston College, Lynch School of Education and Human Development, Chestnut Hill, Massachusetts, United States of America; 6 Centre for Mental Health, University of Rwanda College of Medicine and Health Sciences, Kigali, Rwanda; 7 The World Bank, Washington, DC, United States of America; UFBA: Universidade Federal da Bahia, BRAZIL

## Abstract

Early childhood development (ECD) programmes are heralded as a way to improve children’s health and educational outcomes. However, few studies in developing countries calculate the effectiveness of quality early childhood interventions. This study estimates the cost and cost-effectiveness of the Sugira Muryango (SM) trial, a home-visiting intervention to improve ECD outcomes through positive parent-child relationships. Cost-effectiveness analysis of ECD interventions is challenging given their potential to have multiple benefits. We propose a cost-effectiveness method using a single outcome, in this case the improvement in cognitive development per home-visit session, as an indication of efficiency comparable across similar interventions. The trial intervention cost US$456 per family. This cost will likely fall below US$200 if the intervention is scaled through government systems. The cost-effectiveness analysis suggests that while SM generated a relatively small impact on markers of early development, it did so efficiently. The observed improvements in cognitive development per home-visit are similar to other home-visiting interventions of longer duration. SM by focusing on the family had benefits beyond ECD, including reductions in violence against children and intermate partner violence, further analysis is needed to include these returns in the economic evaluation.

## Introduction

There is general agreement on the importance of the prenatal period and early years of a child’s life in shaping their developmental potential [1]. This period of rapid development is sensitive to environmental influences, with lifelong, possibly irreversible, effects on a person’s wellbeing and productivity [2]. Therefore, interventions that promote positive parent-child interactions and nurturing care and protect against negative experiences in early childhood, such as family violence, have the potential to generate both immediate and long-term returns over the life-course [2–7]. Limited understanding among policy makers of the importance of intervention in the earliest days means that resources aiming to promote early development are often directed to providing centre-based services for older children [8]. While such interventions have the potential to generate significant returns, such returns are unlikely to match those of interventions targeted at the home environment earlier in a child’s life [8, 9].

Home-visiting interventions have emerged as a promising means to intervene in the lives of very young children and their families [10, 11]. Interventions that include a home-visiting component can reach vulnerable hard-to-reach families, and have been linked to long-term outcomes [12, 13]. Home visiting programmes have significant differences in terms of frequency of visits, intervention duration, the levels of training and supervision provided to the home visitors and the inclusion of additional services such as group sessions and food parcels [11, 14, 15]. Interventions of higher intensity, involving frequent visits for extended periods of time, by highly trained staff and the inclusion of other services, require more resources to implement thus may not be affordable in all contexts. Interventions of lower intensity and therefore lower cost may not be sufficient to promote change [16]. There have, however, been encouraging results which suggest that lower intensity, and therefore more affordable, interventions, done by non-specialists, can still be effective [17]. To add to this literature we need to test, cost, and compare lower intensity interventions provided across a range of contexts [18]. Despite this need, home-visiting interventions are seldom subject to cost-analysis, even more so for interventions in LMICs or that address broader features of the home environment such as family functioning and reduction of violence [19, 20]. Moreover, where interventions are costed, it is difficult to effect a comparison given the range and variety of outcomes measured.

The data that are available on the costs of programmes suggests that the resource intensity of ECD interventions varies greatly. Using a standadised costing approach, Verguet et al estimated the cost of home and group based programmes targetting children 0–3 years ranged from $18 to $3,519 [18]. It is clear, therefore, that there is substantial variation in the resource intensity of interventions. What is less clear is how interventions vary in their value for money.

Comparisons of the value for money of interventions can be made using cost effectiveness analysis (CEA) or cost benefit analysis (CBA). CEA requires the selection of a single outcome in order to calculate the cost per unit of that outcome and compare this cost to a selected comparator(s), the cost per outcome being taken as a measure of programme efficiency [21]. This approach has been used to compare ECD interventions when one particular outcome is of primary interest. For example, the cost-effectiveness analysis of 25 home visiting programmes versus usual care for the prevention of child maltreatment (predominantly in the USA) estimated cost-effectiveness as programme cost per case of maltreatment averted [22]. Given, however, that ECD interventions, such as home-visiting interventions, are known to improve multiple outcomes, selecting a single outcome can be problematic. Selecting one outcome will undervalue interventions which improve multiple outcomes compared to those which narrowly focus on the outcome used in the evaluation. Composite outcome measures such as Disability Adjusted Life Years or Quality Adjusted Life Years have been used to address this problem in health care by converting multiple health outcomes into a common unit. They are, however, inadequate here as they include only health benefits and home-visiting interventions for ECD have impacts beyond health [18]. Given these challenges, CBA has at times been preferred to CEA as it allows multiple outcomes to be included in the analysis as long as they are measured in or can be converted to a money metric. Bailey et al, observed that CBA was the preferred approach to economic evaluation across 115 ECD interventions with multiple outcomes [23]. However, they note that interpretation of these results is challenging as it is difficult to attach a monetary value to many of the outcomes associated with ECD interventions [23]. Comparison is further complicated by differences in the CBA methods applied in early childhood programs [7].

In this paper we seek to contribute to the literature on the economic evaluation of ECD interventions in two ways. Firstly, by providing a costing of an effective low intensity home visiting intervention in Rwanda to promote ECD, and secondly by outlining and applying an economic evaluation approach to comparing the relative efficiency of home visiting programmes in LMICs.

Our analysis is of the Sugira Muryango (SM) Intervention and Trial in Rwanda [NCT02510313]. The cost-analysis examines three implementation scenarios to provide an indication of the resource needs of the programme and how these might change if the programme is scaled up and/or adopted by government. The end goal of the SM evaluation is to provide the Rwandan Government with an evidence-based model of the cost and impact of an ECD promotion intervention which could be implemented within their own systems. The approach allows us to examine the relative efficiency of SM. to similar interventions.

We propose a CEA based method using improvements in cognitive development as the measure of intervention effectiveness. We recognise that cognitive development is only one among several important outcomes of early development but suggest that it provides a meaningful indicator of the intervention’s overall impact. If different home visiting interventions improve a similar set of outcomes and the impacts of the intervention on those outcomes are correlated with the impacts on cognitive development, then interventions which more efficiently improve cognitive development can be assumed to be relatively more efficient overall.

We examine cost effectiveness in terms of the returns in terms of cognitive development per home visit, rather than the cost per unit improvement in cognition. We prefer this approach as the differences in costs of home visiting programmes are largely determined by salaries and the ease or difficulty of accessing households. As a result, programme efficiency is conflated with differences in context when using cost per unit but not when using return per visit.

## Methods

### The Sugira Muryango intervention and trial

Sugira Muryango is a home-visiting programme designed to improve early child development and reduce family violence by promoting positive parent-child relationships, nurturing care, problem solving, and shared decision making [24, 25]. SM consists of 12, 60 minute sessions delivered as weekly visits to participants’ homes by trained community based non-specialists over 3 months [26]. The programme services families eligible for social protection through the Rwandan Government’s poverty reduction initiative Vision 2020 Umurenge Programme (VUP). VUP is offered to families in the poorest poverty categorization (Ubedehe 1) and exists in two forms: Classic and extended. Both include cash-for-work, but the extended programme also provides interventions related to financial literacy, asset transfers, skills training and information on health and education. It was envisioned that the additional cash from participation in VUP would complement SM activities through increased material well-being in households.

A cluster-randomized control trial (CRT) was implemented to asses the effectiveness of Sugira Muryango; as well as the interaction between SM and classic/expanded VUP programming and the costs, barriers and facilitators of integrating the SM package into VUP or other government programming, see Table 1 [26]. The study enrolled 1049 VUP-eligible families with children aged 6–36 months from across approximately 52 cells in 3 districts of Rwanda. Families were assigned to one of two treatment or two control arms, designed to test the efficacy of SM implementation alongside both classic and expanded VUP. In the cost and cost effectiveness analysis we pool in the classic and extended VUP participants and examine only control vs intervention. Moderation analyses did not reveal any differential effects by type of VUP program eligibility. Data was collected at baseline, post-intervention, and 12-month follow-up. Analyses were conducted among all children (N = 1,078) or primary and secondary caregivers enrolled at baseline (N = 1,498) under intention to treat (ITT). Attrition rates were low (2.0% for children; 9.6% for caregivers) and there was virtually no differential attrition between treatment and control households. Missing data rates were also low (< 4%) and, where present, missingness is balanced across groups (treatment and control).

**Table 1:** Sugira Muryango CRT arms.

Control Arms	Intervention Arms
**Classic VUP:** Families receive benefits (cash) in exchange for labour-intensive public works.	**Sugira Muryango & Classic VUP:** Families receive a combination of SM and benefits (cash) in exchange for labour intensive public works.
**Expanded VUP:** Families receive benefits (cash) in exchange for flexible public works within close proximity (2 km) to their household. Families are eligible for variation of minimum graduation package benefits, such as asset transfer, financial literacy, skills training, sensitizations.	**Sugira Muryango & Expanded VUP:** Families receive a combination of SM and benefits (cash) in exchange for flexible public works within close proximity (2 km) to their household. Families are eligible for variation of minimum graduation package benefits, such as asset transfer, financial literacy, skills training, sensitizations.

### Ethics statement

The original trial obtained ethical approval from the institutional review boards of the Harvard T. H. Chan School of Public Health and Boston College (IRB16-1570) as well as the Rwanda National Ethics Committee (No. 896/RNEC/2016), and Boston College Institutional Review Board (19.017.04.2). The ClinicalTrials.gov registration is NCT02510313. In the original trial all adult study participants provided written informed consent for their own participation and primary caregivers gave written consent for participation of their children.

### Cost-analysis

We conducted a cost analysis to establish how much a service provider should expect intervention implementation to cost: if they replicate the trial in terms of scale and staffing (scenario 1); if they increase scale to 2000 families every 3 months (scenario 2); and if they increase scale using government systems to 2000 families every 3 months, including government staff (scenario 3). See Tables 2 and 3 for a summary of the scenarios and the key issues related to them.

**Table 2:** Intervention implementation scenarios.

	*Scenario 1- As implemented*	*Scenario 2- Expanded*	*Scenario 3- Government Delivery*
Description	Estimate of the resources used to deliver the intervention trial.	Estimate of the resources used to deliver SM to a large population	Estimate of the resources used to deliver SM through existing government systems.
Considerations	Providing the intervention in the context of a trial is expensive as the start-up costs are not spread out over a long period, or over many beneficiaries. Training costs, for example, are divided only over the number of families receiving the intervention during the trial	Examines the possibility of economies of scale, i.e. the lower unit cost associated with ongoing and larger scale delivery. If SM were run on an ongoing basis, the same trained delivery staff would provide services to many more families, without additional training, and associated costs, thereby reducing the cost per family.	Includes changes in salaries and in the management structure to reflect how SM would be adapted if integrated into existing government systems.

**Table 3:** Assumptions for Phase I scenarios 2 and 3.

	Scenario 1: As implemented	Scenario 2: Expanded	Scenario 3: Government delivery
Assumptions for programme implementation		• Capital costs allocated over their useful life • 3 years until coaches require re-training • 25% improvement in efficiency of coaches with time/experience	• Capital costs allocated over their useful life • 3 years until coaches require re-training • 25% improvement in efficiency of coaches with time/experience • Expenditure on travel and subsistence from head office to implementation site during start-up phase excluded • 70% of transport costs included (i.e. assume 30% for travel to site). • International salaries for management staff adjusted to local rates • Increased rates of supervision • Decreased involvement of senior management
Scale (No. families)	500 once off	2000 every 3 months ongoing	2000 every 3 months ongoing
Families per coach (per cohort)	5.3^a^	6.6^b^	6.6^b^
Coaches per supervisor	10	10	10
Supervisors per manager	15	15	8
Managers per director	0.5	0.5	2

^a^ Equivalent to coaches visiting 4 households per week

^b^ Equivalent to coaches visiting 5 households per week

In each scenario all costs are estimated from a provider perspective and are thus limited to the cost of implementing SA. We do not include the cost carried by families to access these services. As this is a home-visiting programme the financial costs to families are negligible. Families do incur a cost for the time they devote to participating in the program, but these are likely to be small relative to the implementation costs. Moreover, as the end goal is to support government led implementation, the potential budget impact is of primary interest.

#### Staff cost data

SM is a human resource intensive model, therefore, the ratio of beneficiaries and associated sessions to full-time equivalent implementation staff of different levels is the critical feature of the model and was used as the skeleton for all three implementation scenarios. To estimate the costs of implementation under different scenarios using CRT data we follow the same approach as Desmond et al., [27] and Tomlinson et al [28]. The approach differentiates between research and implementation costs, removing the former to allow a focus on the latter. To this end we estimate how many staff would have been required to implement SM if there had been no research conducted. This was done using data from the trial on the number of families recruited, sessions conducted, and staffing levels in Scenario 1. Trial staff were grouped into three categories: implementation only, research only, and staff who worked on both activities. Staff who were involved in research only were not included in the analysis. To estimate the number of full-time equivalent staff needed, the time of staff who were involved in both research and implementation was divided between the two activities. This breakdown was done by the project manager, allocating a percentage to each activity for each month of the project. The monthly allocations allowed for greater precision, as certain months were clearly all implementation and others clearly all research.

This produces the ratio of staff, at different levels, to families served and sessions conducted. We then estimate, from trial records, what expenditure would have been required if no research was conducted. As far as possible we allocate the implementation expenditures to staff categories to give an estimate for the full cost of each staff category. For example, travel and communication costs involved in conducting home visits are allocated to home visitors, while the travel and communication costs associated with supervision are allocated to supervisors and managers. The remaining expenditure items are allocated to overheads. Combining the estimated ratios of families to staff categories with the full cost of each staff category allows us to estimate the cost of delivery at different scales. Moreover, we are able to cost different scenarios by changing either the ratios or the costs of staffing.

Table 3 presents the ratios of families/sessions to coaches (implementation staff), and implementation staff to supervisory staff and supervisory staff to management. The first column of ratios reports the full-time equivalent number of staff, by level, based on the model of implementation employed in the trial. The second and third columns report the adjusted ratios for the other scenarios.

The ratio of families to coaches is based on programme data for scenario 1. For scenarios 2 and 3 it includes the assumed 25% improvement in efficiency. As the programme was implemented in the context of the trial, the project director was heavily involved in implementation to ensure fidelity, allocating more time than the manager. This structure is maintained in scenario 2. However, to provide more realistic estimates for scenario 3, the ratio is changed to 2 (i.e. one director for every two managers). This is still a low ratio to maintain quality. To balance this change, the ratio of supervisors to managers was shifted down from 15 to 8. This was done to allow the assumption that quality can be maintained despite lower levels of involvement from high-level management.

#### Non-staff costs

To estimate resource needs other than staffing, the average resource requirements of training, transport, communication, and other costs per category of staff member was calculated based on an expenditure review. The cost of this resource use, by staff category, was then multiplied by the number of staff in that category in each of the scenarios. This replicates the expenditure data in scenario 1 and provides new estimates for scenarios 2 and 3 based on differences in scale and implementation structure.

The expenditure data used for this exercise was derived from project records by allocating each item to: implementation only, research only or mixed use. An initial review of expenditure data was undertaken by a member of the project staff, who provided a description of each item and indicated the category it should be allocated to. The project manager reviewed the descriptions and allocations, and adjusted as they deemed appropriate, and proposed how to divide mixed costs between research and implementation, based on their understanding of the work involved. Finally, the costing team made the final assessment on the division between research and implementation costs, often in consultation with the project manager, and then allocated to the appropriate level of staff (coaches, supervisors, or management). The costs of training and salaries of implementation staff and providing the necessary communication, transport and consumables, and immediate management support were easily allocated to implementation. Similarly, the costs of data collection were easily excluded. High level management and oversight, along with head office costs were, however, more difficult to allocate between implementation and research. Where in doubt, we allocated costs to implementation to be conservative.

For scenario 1, capital costs were fully allocated in the period in which they occurred, unless they were likely to have value at the end of the project (e.g. vehicles). For scenarios 2 and 3 they were allocated based on their expected useful life. For a summary of the capital costs and assumptions see S4–S6 Tables.

The expenditure review provided an estimate of the full cost of each staff level, broken down into the following categories: training, salary, materials, transport, communications, office, and cost of employment. It also provided an estimate of indirect administrative costs, which could not be allocated by staff category, and are included as an overhead.

#### Costing the scenarios

To estimate of the cost of implementation for each scenario, the full cost of each staffing category (salary plus expenditure allocation) was multiplied by the number of staff required in each category and indirect cost were added. This cost was then divided by the number of families covered and the number of sessions provided. This allows us to report the cost per family covered and the cost per session. The data from the trial on staffing needs and expenditure were used to estimate scenario 1. For scenarios 2 and 3, changes were made to the staff ratios or the cost of inputs to reflect what we assume will happen with changes in scale and delivery mechanisms. These changes are summarised in Table 3.

### Cost-effectiveness analysis

The CRT of SM examined consequences of the intervention on multiple diverse outcomes at 3 months post intervention [26]. Such a range of benefits frustrates the use of cost effectiveness analysis if calculating cost per unit of outcome. To address this challenges, we selected one SM outcome, child cognitive development, and compared improvements in this outcome per home visit across interventions implemented in similar contexts that included a home-visiting component and demonstrated an improvement in child cognitive outcomes summarized in S1 Table. Cognitive development was measured using different tools across different studies, therefore to facilitate comparison, improvements to child cognition have been converted to standard deviations.

We selected cognitive development because it is a critical outcome, is widely available in evaluations of ECD interventions and is likely correlated with other desired outcomes, given common pathways to impact. In early life multiple interactive developmental processes occur simultaneously, setting the foundation for human development across the life-course. Commonly measured outcomes of early childhood development include: cognition, language, motor skills, social-emotional development, and psychosocial wellbeing/ mental health [18, 29, 30]. These domains are sensitive to common risk and protective factors [31, 32] targeted by early interventions such as the provision of nurturing care [2]. The mitigation of common risks and promotion of common protections should improve multiple domains of early development. A further advantage of using early cognition as a lead indicator is its linkage to multiple short and long-term benefits [33, 34]; and bi-directional relationships with early motor skills [35], emotional capacities [36], and adolescent well-being [37]. CEA typically calculate the cost per unit of outcome (cost/outcome), whereas we calculate the return per session (outcome/no. of sessions). Our approach allows us to investigate relative efficiency of interventions implemented across countries, independent of differences in country level costs and contextual cost drivers. Essentially, we are interested in the return in programme efficiency understood as on improvements in cognitive development per session, rather than the return on the money spent on the intervention, as the latter will be largely determined by context. S2 and S3 Tables detail the intervention impacts across multiple domains in the selected comparison studies.

For interventions which included group and home sessions, we include an estimate of the relative resource use of a group vs a home session. To estimate how much less resource intense per participant a group session is compared to a home-visit we drew upon an RCT in rural India which found the group sessions are significantly less costly per participant (home visiting: USD$135 vs group sessions USD$38) but of similar impact on child cognitive and language outcomes (home visiting: 0.324 SD, 95% confidence interval [CI]: 0.152 to 0.496, P = .001 vs group sessions: 0.281 SD, 95% CI: 0.100 to 0.463, P = .007) [38]. Therefore we report the group sessions as 0.2 a home visit and as 0.5 a home visit.

The methods used are compliant with the The Consolidated Health Economic Evaluation Reporting Standards (CHEERS) statement as reported in S7 Table [39].

## Results

### Cost analysis

The costs per family covered and per session and the breakdown of costs by expenditure category are summarised in Table 4, for each of the 3 scenarios. Staff costs are largest in all the scenarios.

**Table 4:** Cost per family and per session, and proportion of costs by expenditure category, by scenario.

	Scenario 1: As implemented	Scenario 2: Expanded.	Scenario 3: Government delivery
**Cost per family**	$456	$262	$199
**Cost per session**	$38	$22	$17
**Proportion of costs ($ value)**	(500 families once off)	(2000 families every 3 months)	(2000 families every 3 months)
**Salaries**	0.52 ($118,560)	0.64 ($335,360)	0.59 ($234,820)
**Training**	0.24 ($54,720)	0.04 ($20,960)	0.05 ($19,900)
**Communication**	0.02 ($4,560)	0.03 ($15,720)	0.04 ($15,920)
**Transport**	0.16 ($36,480)	0.21 ($110,040)	0.22 ($87,560)
**Office costs**	0.02 ($4,560)	0.04 ($20,960)	0.06 ($23,880)
**Overhead**	0.04 ($9,120)	0.04 ($20,960)	0.04 ($15,920)
**Total**	$228,000	$524,000	$398,000

### Cost effectiveness

Table 5 reports the estimated impact on cognitive development per session. The impact is measured in standard deviation improvements and the number of sessions is based on the protocol for each study.

**Table 5:** Impact on cognitive development per home visiting session.

Study	Intervention	Country	Measure of cognition	Standardised impact on cognitive development (Standard deviation)	No. of home visits/group sessions (intended)	Impact per home-visit (Ex. Group sessions)	Impact per home-visit (Group sessions weighted 0.2)	Impact per home-visit (Group sessions weighted 0.5)
	Sugira Muryango	Rwanda	ASQ-3	0.11	12/0	0.009	-	-
Hamadani et al., 2006	Centre based nutrition supplementation + psychosocial stimulation	Bangladesh	BSID-III: Mental Development Index score	0.33	80/44	-	0.004	0.003
Eickmann et al., 2003	Psychosocial stimulation	Brazil	BSID-II Mental Development Index score	0.5	11/3	-	0.043	0.040
Attanasio et al., 2014	Psychosocial stimulation	Colombia	BSID-III cognitive scale raw score	0.260	78/0	0.003	-	-
Grantham-McGregor et al.; 2020	Psychosocial stimulation + nutritional education	India	ASQ-3 problem solving (baseline)	0.324	96/0	0.003	-	-
			BSID-III cognition (Endline)					
Gardner et al., 2005	Psychosocial stimulation	Jamaica	Griffiths Mental Development Scales: Performance subscale	0.22^a^	24/0	0.009	-	-
Powell et al., 2004	Psychosocial stimulation	Jamaica	Griffiths Mental Development Scales: Performance subscale	0.86^a^	50/0	0.017	-	-
Lopez Garcia et al., 2021; Luoto et al., 2021	Psychosocial stimulation + nutrition education	Kenya	BSID-II scaled cognitive scores	0.34	4/12	-	0.053	0.034
Yousafzai et al., 2014	Psychosocial stimulation	Pakistan	BSID-III cognitive scale composite score	0.6	24/24	-	0.021	0.017
Caridad-Araujov et al., 2021.	Psychosocial stimulation	Peru	ASQ-3 Cognitive development	0.022^b^	10/0	0.002	-	-

^a^ (Zhang et al., 2021)

^b^ Treatment on the treated

## Discussion

The costs of the SM home-visiting intervention are within the range reported in the ECD literature. Costs per family for ECD interventions for under 3’s range from $18 to $3,519 [18]. In all three scenarios the largest expenditure category is salaries, followed by transport costs, given that this is a human resource intensive intervention.

Scenario 1 is the most costly scenario per family. The higher costs stemming from the allocation of start-up costs to a few families and salaries of international staff. In Scenario 2 and 3 the cost per family is substantially reduced, reflecting the potential for economies of scale, and because we assume some improvement in efficiency associated with learning and enrolled households being closer together and so moving between them takes less time. If the assumption of efficiency is not made, the costs per family would be US$40 higher in both scenarios. The costs here are based on a programme delivered in rural areas, thus it is likely that there will be substantial cost savings in urban settings where households are closer together, allowing coaches to conduct more sessions in a week and lowering transport costs. For example, assuming coaches could visit 10 families a week in urban settings would lower the cost per family by close to $100 in scenarios 2 and 3.

The cost-analysis results for scenario 3 suggest that incorporating SM in its current structure into Government systems in rural areas would be associated with a further drop in the cost per family. This is a result of lower management costs associated with the shift from international to local management. It is important to note here that the current estimate for scenario 3 includes a payment to the coaches, which is not the norm in Rwanda. Removing this payment drops the cost by $20 per family (approximately 10%). However, the quality of the intervention may not be maintained if the coaches are not salaried.

The cost-effectiveness results when looking only at the cognitive development resulting from SM are relatively small, however, the intervention is nonetheless comparable, in cost effectiveness terms, to several other similar interventions of longer duration. SM involves far fewer sessions than similar interventions. Essentially, the SM intervention delivers a smaller dosage of home-visits but still leads to improved outcomes suggesting the potential of low intensity home-visiting programs to improve cognitive development.

The improvement in cognitive development in SDs. per session is 0.009, the same as the return seen to intervention in Jamaica (0.009 home visits only); more than interventions in Peru (0,002 home visits only); Colombia (0.003 home visits only); India (0.003 home visits only); Bangladesh (0.003 home visits with group sessions weighted 0.5); and less than other interventions in Jamaica (0.017 home visits only); Pakistan (0.017 home visits with group sessions weighted 0.5); Kenya (0,034 home visits with group sessions weighted 0.5); and Brazil (0.040 home visits with group sessions weighted 0.5) [14, 17, 38, 40–46].

It is noteworthy that many of the studies which performed well in the cost effectiveness analysis included group sessions. Moreover, the Msingi Bora trial included an arm with only groups which they estimated led to a return of $15.5 for every $1 invested. These results suggest that group-based components may be efficient additions, or substitutes, for home visiting. Indeed, a recently evaluated group-based intervention trial in Rwanda had a successful impact on child cognitive outcomes [47]. Zhang et al noted that “for the cognitive development domain, the effect sizes were greater for interventions delivered through group sessions compared to individual sessions (ES = 0.53 vs. 0.28, Q = 4.99, p = 0.03)” [48]. However, a recent systematic review of parenting interventions to improve ECD and parent outcomes found there to be no statistically significant subgroup results by intervention setting be it home visit, clinic or community; thus, while parenting groups may prove less costly in some circumstances, a careful consideration of the context and the population served must inform the final design [49].

A cost-effectiveness study of an intervention similar to SM is that of the Pakistan Early Child Development Scale-Up (PEDS) trial, results of the intervention are included in Table 4 [19]. The PEDS trial integrated responsive stimulation and nutrition programs (alone or in combination) into an existing health care programme. It involved home visits and group meetings to improve child cognitive, language, and motor development. The responsive stimulation component was costed at US$4 per child per month when integrated within the existing community health programme for parents of children below the age of 2 years. As the PEDS visits occurred monthly, the cost per child per month is comparable to the SM cost per session, if also delivered monthly. To examine cost effectiveness, Gowani et al. divided the annualised cost of delivery staff by the average cognitive score of children in that arm [19]. The comparison suggests the PEDS intervention was less costly both per session and overall than SM. They found the combination (stimulation and nutrition) intervention to be most cost effective of the arms. While the PEDS CEA approach allows for comparisons between the study arms, it does not lend itself to effectiveness comparisons with other interventions because of differences in baseline results and measures of cognition, nor does it accommodate delivery cost differences within and across countries.

Verguet et al., recently suggested an analytical framework for evaluating ECD interventions to address variations in costs between countries they used standardised unit costs [18]. The advantage of Verguet et al.,’s approach is that it accounts for variations in the qualification levels of staff across programmes; not capture in approach of examining the cost per session. The disadvantage is that Verguet et al., do not account for differences in context which have an impact on the cost of delivery. For example, our approach allows the direct comparison of urban and rural programmes, while their approach would suggest a lower cost for the urban programme, given ease of delivery, which risks being interpreted as model efficiency. Our approach excludes variations in cost associated with the use of existing infrastructure. Verguet and colleagues found that a programme which was able to make use of the existing systems was the most cost effective, but this is not helpful for contexts without such infrastructure [18]. As an outcome measure, they similarly use standardised outcomes, but whereas we use only cognitive outcomes, they measure early childhood development through a weighted average of improvements in motor, language, socio-emotional and cognitive development. While this provides a more inclusive framework for evaluation, it is not clear how the weights should be determined. In their reported results they weigh outcomes equally. Two studies costed by Verguet et al., also included in this paper used home-visiting to effect changes in child development in Jamaica [40, 44]. Dividing the standardised cost Verguet et al., present for each of these studies per child by the number of sessions delivered in the intervention costs one intervention at $53,42 per home visit delivering nutrition and stimulation or stimulation only [40] and the other at $24,8 per home visit delivering stimulation only [44]. The cost of the stimulation only programme at $24,8 dollars per home visit, as integrated into the primary health care system, is comparable with the $22 cost per visit of SM in Scenario 2.

Our analysis has focused on determining the relative cost efficiency of SM as an ECD intervention. However, it is important to note that some of the mechanisms through which the intervention aims to improve ECD outcomes have an intrinsic value. Most notably, the intervention aims to reduce family violence, including IPV. Focusing only on the ECD outcomes does not fully value reductions in violence and highlights the risks a narrow evaluative frame. To address this, improvements in mechanisms which have intrinsic value can be reported alongside cost effectiveness results to ensure policy makers are fully informed.

Our analysis has several limitations. The cost analysis for scenarios 2 and 3 is modelled. While it is informed by data from the trial, it nonetheless relies on assumptions and further work to monitor costs as scale is increased will be important. By using a single outcome to measure improvements in ECD, the assessment of relative efficiency is dependent on the extent to which the assumptions that cognitive development is correlated with other outcomes and that those other outcomes are similar across interventions holds. If SM or other interventions have outsized effects on outcomes other than cognitive development the analysis will lead to an under or overestimate of the relative efficiency of SM, respectively. This is an important consideration for SM given its focus on violence in addition to ECD. Families who received SM exhibited a decreased in harsh discipline (incidence rate ratio, IRR = 0.741, 95% CI 0.657 to 0.835) and intimate partner violence (IRR = 0.616, 95% CI:0.458 to 0.828) [27]. These violence outcomes are not included in the analysis but should be considered by policy makers when assessing the value for money of the intervention.

The SM trial, costing and CEA were conducted primarily to inform policy discussions within the Government of Rwanda. To this end, our cost analysis shows that while the intervention was fairly expensive when delivered as part of a trial, there are clear opportunities to reduce the costs, firstly by increasing the scale of intervention and secondly by integrating delivery with government services. The CEA shows that the impact per session is comparable to other effective interventions. This suggests that if the government only has funds to fund a relatively short intervention, it will still have an impact. It also suggests that if more funds are available, additional benefits should result from additional sessions. While the focus on outcomes per session avoids having to deal with differences in costs associated with differences in context, it effectively assumes that the sessions of different interventions could be implemented for similar costs if they were implemented in the same context. This ignores the possibility that some programmes may have more highly trained or supervised staff.

In conclusion, we found that SM provides a model of home visiting for a resource constrained context. When looking only at cognitive development as a comparable outcome, the overall impact is relatively small but achieved efficiently. Our results suggest that the impact may be magnified by the inclusion of costing of the VAC and IPV results. Group sessions may also enhance the benefits of the programme. We note that it is difficult to project what impacts improvements in domains such as improved communication, social skills, and emotional wellbeing may have across the lifespan for an individual, their family, their community, and the economy. The real benefits to the individual and society may be significantly greater than what we are able to quantify.

## Supporting information

S1 Table Comparison of interventions with a home-visiting and psychosocial stimulation component with positive early cognitive outcomes. (DOCX)

S2 Table Comparison of developmental outcomes included in interventions. (DOCX)

S3 Table Selected interventions reported standardised impact on development. (DOCX)

S4 Table Assumptions applied to expenditure data. (DOCX)

S5 Table Capital costs average useful life. (DOCX)

S6 Table Costing inputs. (DOCX)

S7 Table Consolidated Health Economic Evaluation Reporting Standards 2022 (CHEERS 2022) checklist. (DOCX)
