# Supplementary material for: Measuring the cost-effectiveness of a home-visiting intervention to promote early child development among rural families linked to the Rwandan social protection system
Source: PLOS Glob Public Health. 2023 Oct 24;3(10):e0002473. doi: 10.1371/journal.pgph.0002473 (PMC10597512; doi:10.1371/journal.pgph.0002473)
Supplement: S1 Table — (DOCX) [file pgph.0002473.s001.docx]

**S1 Table. Comparison of interventions with a home-visiting and psychosocial stimulation component with positive early cognitive outcomes**

| **Author** | **Country** | **N** | **Study population** | **Intervention** | **Frequency of home-visits, duration & delivery** | **Intervention duration** | **Measure** | **Cognitive outcomes**  **(mean ± SD)** |
| --- | --- | --- | --- | --- | --- | --- | --- | --- |
| Sugira Muryango | Rwanda | 2911 | 6-36mo, VUP eligible (very low-SES) | Treatment: Psychosocial stimulation & conflict resolution and problem solving/resource navigation through home-visits  Classic or Expanded VUP public works (control)  Combined Classic or Expanded VUP public works with Sugira Muryango (treatment) | Weekly, 90mins, Non-professional | 3–4  month | ASQ-3 | Sugira Muryango + PW (n=537)  Baseline 37.2± 16.0  Endline 39.7± 22.9  PW only (n=507)  Baseline 35.6 ±16.1  Endline 38.0 ±15.2 |
| Hamadani *et al.* (2006)^1^ | Bangladesh | 299 | 6-24mo  Undernourished control  Undernourished intervention  Better nourished comparison | Centre based nutrition supplementation +  Psychosocial stimulation through home-visits + group meetings | Twice weekly (first 8mo) to weekly (last 4mo), Non-professional | 12mo | BSID-III: Mental Development Index score | Undernourished intervention (n=92)  Baseline 89.4 ±15.0 / endline 87.6 ±13.3  Undernourished control (n=101)  Baseline 90.1± 15.1 / endline 82.7 ±17.2  Better nourished comparison (n= 106)  Baseline 93.1±13.5/ endline 89.3 ± 13.1 |
| Eickmann *et al.* (2003)^2^ | Brazil | 156 | 12mo, Low-SES | Psychosocial stimulation through initial home visit, 3 workshops, and 10 reinforcement home visits. | Weekly, 30-45mins, Home-visitors (non-professional) | 5mo | BSID-II Mental Development Index score | Control (n=78)  12mo 101.9±10.7 / 18mo 95.6 ±13.7  Intervention (n=78)  12mo 101.2±11.0 / 18mo 105±8.8 |
| Attanasio *et al.* (2014)^3^ | Colombia | 1420 | 12-24mo, Low-SES | Micronutrient supplementation only.  Stimulation through home-visits only.  Supplementation + stimulation. | Weekly, Female community leaders (non-professional) | 18mo | BSID-III cognitive scale raw score | Control (n=318)  Baseline 52.21 ±7.72 /Endline 71.68 ±4.38  Stimulated (n=318)  Baseline 51.76 ±7.79 /Endline 72.74±4.31  Supplemented (n=308)  Baseline 51.78 ±7.37 /Endline 71.63  ±4.26  Supplemented & stimulated (n=319)  Baseline 51.83 ±7.41 /Endline 72.43±4.27 |
| Grantham-McGregor (2020)^4^ | India | 1449 | 7-12mo | Nutritional education  Nutritional education and home visiting (psychosocial stimulation)  Nutritional education and group sessions (psychosocial stimulation)  Control | Weekly, ~60mins, Trained local women | 24mo | ASQ-3 problem solving (baseline)  BSID-III cognition (Endline) | **Estimated coefficients are expressed in SDs of the control group**  Baseline to endline  Nutritional education (n=1298)  0.037  Home visits and nutritional education (n=1298)  0.324  Group sessions and nutritional education (n=1298)  0.281 |
| Gardner et al., **(**2005)**^5^** | Jamaica | 114 | 9-30mo, Undernourished children | Stimulation through home-visit play session only.  Zinc supplementation.  Zinc supplementation + stimulation.  Control (routine care only). | Weekly, 30min-1hr, Community health aide (para-professional) | 6mo | Griffiths Mental Development Scales: **Performance score** | **Supplemented + stimulated (n=25)**  Baseline 93.8 ± 12.0 /6mo 92.4 ± 16.7  **Stimulated (n=21)**  Baseline 99.7 ± 13.0/6mo 91.3 ± 11.2  **Supplementation (n=30)**  Baseline 95.7 ± 13.6/6mo 86.5 ± 12.2  **Control (n=38)**  Baseline 98.6 ± 15.2/6mo 88.6 ± 12.2 |
| Powell et al., (2004)^6^ | Jamaica | 129 | 9-30mo | Stimulation through home-visit play session.  Control. | Weekly, 90mins, Community health aide (para-professional) | 12mo | Griffiths Mental Development Scales: **Performance score** | **Intervention (n=65)**  Baseline 99.6±12.7/ Follow up 94.5±15.3  **Control (n=64)**  Baseline 98.6±13.1/ Follow up 83.0±10.9 |
| Lopez Garcia *et al.* (2021)^7^ &  Luoto *et al.* (2021)8 | Kenya | 1070 | 6-24mo, Rural | Responsive stimulation +  nutrition education through parenting groups only.  Responsive stimulation +  nutrition education through group sessions + home visits. | Every 8 weeks, 1hr, Community Health Volunteers (non-professional) | 8mo | BSID-II scaled cognitive scores | Control (n=351)  Baseline 9.5±2.3 / Endline 8.74±1.48  Group-only (n=346)  Baseline 9.3±223 / Endline 9.45 ±1.73  Mixed-delivery (n=373)  Baseline 9.5±2.3/ Endline 9.10±1.44 |
| Yousafzai *et al.* (2014)^9^ | Pakistan | 1411 | <2.5mo ,Low-SES | Responsive stimulation only.  Enhanced nutrition only.  Responsive stimulation +nutrition. | Monthly, 30min, Lady Health Workers (non-professional) | 24mo | BSID-III cognitive scale composite score | Responsive stimulation intervention (n=696 at 12 mo, n=701 at 24 mo)  12mo 97.1 ±14.2/ 24mo 81.7 ±14.7  No responsive stimulation intervention (n=661 at 12 mo, n=680 at 24 mo)  12mo 92.0 ±13.0 / 24mo 74.1 ±13.5  Enhanced nutrition intervention  (n=658 at 12 mo, n=676 at 24 mo)  12mo 95.9±12.7 / 24mo 78.4 ±14.6  No Enhanced nutrition intervention (n=699 at 12 mo, n= 705 at 24 mo)  12mo 93.4 ±14.8 / 24mo 77.6 ±14**.**7 |
| Caridad Araujov *et al.* (2021)^10^ | Peru | 4685 | 0-24mo, High levels of poverty and stunting | Psychosocial stimulation via play-based home visits | Weekly, 1 hr, Paraprofessionals | 24mo | ASQ-3 (Cognitive development) | Not reported |

^1^ Hamadani JD, Huda SN, Khatun F, Grantham-mcgregor SM. Psychosocial stimulation improves the development of undernourished children in rural Bangladesh 1. J Nutr Ingestive Behav Neurosci. 2006; 2645–2652.

^2^ Eickmann SH, Lima AC V, Guerra MQ, Lima MC, Lira PIC, Huttly SRA, et al. Improved cognitive and motor development in a community-based intervention of psychosocial stimulation in northeast Brazil. Dev Med Child Neurol. 2003;45: 536–541. doi:10.1017/s0012162203000987.

^3^ Attanasio OP, Fernández C, Fitzsimons EOA, Grantham-mcgregor SM, Meghir C, Rubio-codina M. Using the infrastructure of a conditional cash transfer program to deliver a scalable integrated early child development program in Colombia : cluster randomized controlled trial. BMJ. 2014;349: g5785. doi:10.1136/bmj.g5785.

^4^ Grantham-McGregor S, Adya A, Attanasio O, Augsburg B, Behrman J, Caeyers B, et al. Group sessions or home visits for early childhood development in India: a cluster RCT. Pediatrics. 2020;146. doi: 10.1542/peds.2020-002725.

^5^ Gardner JMM, Powell CA, Baker-Henningham H, Walker SP, Cole TJ, Grantham-McGregor SM. Zinc supplementation and psychosocial stimulation: effects on the development of undernourished Jamaican children–. Am J Clin Nutr. 2005;82(2):399–405.

^6^ Powell C, Baker-Henningham H, Walker S, Gernay J, Grantham-McGregor S. Feasibility of integrating early stimulation into primary care for undernourished Jamaican children: cluster randomised controlled trial. BMJ. 2004;329: 89.

^7^ Lopez Garcia I, Saya UY, Luoto JE. Cost-effectiveness and economic returns of group-based parenting interventions to promote early childhood development: Results from a randomized controlled trial in rural Kenya. PLOS Med. 2021;18: e1003746. doi:10.1371/journal.pmed.1003746.

^8^ Luoto JE, Garcia IL, Aboud FE, Singla DR, Fernald LCH, Pitchik HO, et al. Group-based parenting interventions to promote child development in rural Kenya: a multi-arm, cluster-randomised community effectiveness trial. Lancet Glob Heal. 2021;9: e309–e319.

^9^ Yousafzai AK, Rasheed MA, Siyal S. Integration of parenting and nutrition interventions in a community health program in Pakistan:an implementation evaluation. Ann N Y Acad Sci. 2018;1419: 160–178. doi:10.1111/nyas.13649.

^10^ Caridad Araujov M, Dormal M, Grantham-McGregor S, Lazarte F, Rubio-Codina M, Schady N. Home visiting at scale and child development. J Public Econ Plus. 2021;2: 100003. doi: 10.1016/j.pubecp.2021.100003**S3**
