# Supplementary material for: Measuring the cost-effectiveness of a home-visiting intervention to promote early child development among rural families linked to the Rwandan social protection system
Source: PLOS Glob Public Health. 2023 Oct 24;3(10):e0002473. doi: 10.1371/journal.pgph.0002473 (PMC10597512; doi:10.1371/journal.pgph.0002473)
Supplement: S2 Table — (DOCX) [file pgph.0002473.s002.docx]

**S2 Table. Comparison of developmental outcomes included in interventions**

| **Author, Country** | **Cognitive measure used in comparison** | **Motor measure & outcomes**  **(mean ± SD)** | **Language measure & outcomes**  **(mean ± SD)** | **Socio-emotional Measure & outcomes**  **(mean ± SD)** |
| --- | --- | --- | --- | --- |
| Sugira Muryango; Rwanda | ASQ-3  Sugira Muryango + PW (n=537)  Baseline 37.2± 16.0  Endline 39.7± 22.9  PW only (n=507)  Baseline 35.6 ±16.1  Endline 38.0 ±15.2 | **ASQ-3 Gross motor raw score & Fine motor Raw score mean endline baseline differences**  Sugira Muryango + PW (n=537)  *Gross motor*  46,63± 13,62  *Fine motor*  25,88± 16,27  PW only (n=507)  *Gross motor*  44,90±14,02  *Fine motor*  25,30±16,23 | **ASQ-3 communication raw score mean endline baseline differences**  Sugira Muryango + PW (n=537)  46,60± 15,98  PW only (n=507)  44,03±16,55 | **ASQ-3 social emotional raw score mean endline baseline differences**  Sugira Muryango + PW (n=537)  41,19± 41,19  PW only (n=507) |
| Hamadani *et al.* (2006)^1^; Bangladesh | **BSID-III: Mental Development Index (MDI) score**  **Undernourished intervention (n=92)**  Baseline 89.4 ±15.0 / endline 87.6 ±13.3  **Undernourished control (n=101)**  Baseline 90.1± 15.1 / endline 82.7 ±17.2  **Better nourished comparison (n= 106)**  Baseline 93.1±13.5/ endline 89.3 ± 13.1 | **Bayley II Psychomotor development Index (PDI) score**  **Undernourished intervention (n=92)**  Baseline 82.9±17.2 / Endline 93.0±16.4  **Undernourished control (n=101)**  Baseline 86.6±16.9 / Endline 90.2±17.4  **Better nourished comparison (n= 106)**  Baseline 94.8±12.9 / Endline 99.5±15.5 | - | **Behaviour ratings**  **Undernourished intervention (n=92)**  *Responsiveness*  Baseline 6.3±1.2 / Endline 6.0±1.0  *Emotional tone*  Baseline 5.4±1.6/ Endline 5.5±1.1  *Activity*  Baseline 4.8±1.1 / Endline 4.2±1.2  *Cooperation*  Baseline 5.7±1.6/ Endline 5.6±1.1  *Vocalization*  Baseline 3.7±1.9/ Endline 4.4±1.7  **Undernourished control (n=101)**  *Responsiveness*  Baseline 6.0±1.3/ Endline 5.5±1.1  *Emotional tone*  Baseline 5.6±1.4 / Endline 5.2±1.2  *Activity*  Baseline 4.6±1.1 / Endline 4.2±1.3  *Cooperation*  Baseline 5.9±1.6/ Endline 5.2±1.1  *Vocalization*  Baseline 4.0±1.6/ Endline 4.0±1.8  **Better nourished comparison (n= 106)**  *Responsiveness*  Baseline 6.3±1.4 / Endline 5.8±1.2  *Emotional tone*  Baseline 5.7±1.3/ Endline 5.8±1.2  *Activity*  Baseline 4.6±1.2/ Endline 4.4±1.3  *Cooperation*  Baseline 5.9±1.4/ Endline 5.8±1.3  *Vocalization*  Baseline 4.1±2.1 / Endline 4.8±1.6 |
| Eickmann *et al.* (2003)^2^; Brazil | **BSID-II Mental Development Index (MDI) score**  **Control (n=78)**  12mo 101.9±10.7 / 18mo 95.6 ±13.7  **Intervention (n=78**)  12mo 101.2±11.0 / 18mo 105±8.8 | **Bayley II Psychomotor development Index (PDI) score**  **Control (n=78)**  12mo 100.4±13.1 / 18mo 96.6±13.6  **Intervention (n=78)**  12mo 101.0 ±11.7 / 18mo 104.8 ±11.3 | - | - |
| Attanasio *et al.* (2014)^3^  Colombia | **BSID-III cognitive scale raw score**  **Control (n=318)**  Baseline 52.21 ±7.72 /Endline 71.68 ±4.38  **Stimulated (n=318)**  Baseline 51.76 ±7.79 /Endline 72.74±4.31  **Supplemented (n=308)**  Baseline 51.78 ±7.37 /Endline 71.63  ±4.26  **Supplemented & stimulated (n=319)**  Baseline 51.83 ±7.41 /Endline 72.43±4.27 | **Bayley III**  **Control (n=318)**  *Fine motor*  Baseline 34.85±3.96 /Endline 50.38±4.65  *Gross motor*  Baseline 50.52±6.91 /Endline 63.31±2.79  **Stimulated (n=318)**  *Fine motor*  Baseline 34.64±4.01/Endline 50.77±4.45  *Gross motor*  Baseline 51.11± 7.19/Endline 63.30±2.77  **Supplemented (n=308)**  *Fine motor*  Baseline 34.29±4.07/Endline 50.52±4.56  *Gross motor*  Baseline 50.54±6.34/Endline 63.19±2.99  **Supplemented & stimulated (n=319)**  *Fine motor*  Baseline 34.08 (3.89) ±/Endline ±50.59 (4.50)  *Gross motor*  Baseline ±50.23 (6.90)/Endline ±62.97 (3.08) | **Bayley III**  **Control (n=318)**  *Receptive language*  Baseline 20.57±4.99 /Endline 34.06±3.55  *Expressive language*  Baseline 20.48±6.30/Endline 35.97±5.43  **Stimulated (n=318)**  *Receptive language*  Baseline 20.51±5.00/Endline 34.81*± 3.56  *Expressive language*  Baseline 20.57±6.69/Endline 36.32±5.70  **Supplemented (n=308)**  *Receptive language*  Baseline 20.20±5.23/Endline 34.06±3.73  *Expressive language*  Baseline 19.96±6.18/Endline 36.13±5.85  **Supplemented & stimulated (n=319)**  *Receptive language*  Baseline 19.97±4.77/Endline 34.48±3.43  *Expressive language*  Baseline 19.97±6.14/Endline 36.10±5.81 | - |
| Grantham-McGregor (2020)^4^; India | **ASQ-3 problem solving (baseline)**  **BSID-III cognition (Endline)**  **Estimated coefficients are expressed in SDs of the control group**  Baseline to endline  **Nutritional education (n=1298)**  0.037  **Home visits and nutritional education (n=1298)**  0.324  **Group sessions and nutritional education (n=1298)**  0.281 | **Bayley III**  **Estimated coefficients are expressed in SDs of the control group \| Baseline to endline**  **Nutritional education (n=1298)**  0.075  **Home visits and nutritional education** (n=1298)  0.055  **Group sessions and nutritional education** (n=1298)  0.144 | **Bayley III**  **Estimated coefficients are expressed in SDs of the control group \| Baseline to endline**  **Nutritional education (n=1298)**  0.127  **Home visits and nutritional education** (n=1298)  0.239  **Group sessions and nutritional education** (n=1298)  0.302 | **-** |
| Gardner et al., **(**2005)^5^; Jamaica | **Griffiths Mental Development Scales: Performance score**  **Supplemented + stimulated (n=25)**  Baseline 93.8 ± 12.0 /6mo 92.4 ± 16.7  **Stimulated (n=21)**  Baseline 99.7 ± 13.0/6mo 91.3 ± 11.2  **Supplementation (n=30)**  Baseline 95.7 ± 13.6/6mo 86.5 ± 12.2  **Control (n=38)**  Baseline 98.6 ± 15.2/6mo 88.6 ± 12.2 | **Griffiths Mental Development Scales: Locomotor & Hands and eyes score**  **Supplemented + stimulated (n=25)**  *Locomotor score*  Baseline 106.6±9.6 /6mo 101.8±10.7  *Hand eye coordination*  Baseline 103.7 ± 9.2/6mo 102.0 ± 9.4  **Stimulated (n=21)**  *Locomotor score*  Baseline 106.9±11.1 /6mo 98.6 ±11.8  *Hand eye coordination*  Baseline 107.2 ± 8.9/6mo 94.6 ± 8.8  **Supplementation (n=30)**  *Locomotor score*  Baseline 105.1±15.3 /6mo 97.4±12.7  *Hand eye coordination*  Baseline 100.9 ± 12.6/6mo 95.3 ± 11.2  **Control (n=38)**  *Locomotor score*  Baseline 112.1±16.6 /6mo 102.6±9.9  *Hand eye coordination*  Baseline 108.3 ± 13.3/6mo 98.3±11.3 | **Griffiths Mental Development Scales: Hearing and speech score**  **Supplemented + stimulated (n=25)**  Baseline 105.3 ± 12.2 /6mo 100.4 ±11.5  **Stimulated (n=21)**  Baseline 108.1 ± 9.8 /6mo 100.4 ± 16.0  **Supplementation (n=30)**  Baseline 102.1 ± 14.7 /6mo 89.1 ± 11.1  **Control (n=38)**  Baseline 107.5 ± 17.0 /6mo 96.6 ± 13.9 | - |
| Powell et al., (2004)^6^; Jamaica | **Griffiths Mental Development Scales: Performance subscale**  **Intervention (n=65)**  Baseline 99.6±12.7/ Follow up 94.5±15.3  **Control (n=64)**  Baseline 98.6±13.1/ Follow up 83.0±10.9 | **Griffiths Mental Development Scales: Locomotor & Hands and eyes score**  **Intervention (n=65)**  *Locomotor score*  Baseline 108.7±11.0/ Follow up 104.2±12.2  *Hand and eye*  Baseline106.8±9.7/ Follow up 97.6±10.7  **Control (n=64)**  *Locomotor score*  Baseline 108.5±12.6/ Follow up 102.1±14.2  *Hand and eye*  Baseline 104.9±11.4/ Follow up 90.6±8.9 | **Griffiths Mental Development Scales: Hearing and speech score**  **Intervention (n=65)**  Baseline 106.3±11.7/ Follow up 100.7±15.4  **Control (n=64)**  Baseline 104.7±13.7/ Follow up 89.4±13.9 | - |
| Lopez Garcia *et al.* (2021)^7^ & Luoto *et al.* (2021)^8^; Kenya | **BSID-II scaled cognitive scores**  **Control (n=351)**  Baseline 9.5±2.3 / Endline 8.74±1.48  **Group-only (n=346)**  Baseline 9.3±2.2 / Endline 9.45 ±1.73  **Mixed-delivery (n=373**)  Baseline 9.5±2.3/ Endline 9.10±1.44 |  | **BSID-II language**  **Control (n=351)**  *Receptive language*  Baseline 9.7±2.3/ Endline 9.75±1.75  *Expressive language*  Endline 8.85±1.74  **Group-only (n=346)**  *Receptive language*  Baseline 9.3±2.2/ Endline 10.46±2.23  *Expressive language*  Endline 8.92±1.71  **Mixed-delivery (n=373**)  *Receptive language*  Baseline 9.6±2.1/ Endline 10.12±1.78  *Expressive language*  Endline 8.64±1.61 | **Raw Wolke socioemotional score, 7–35**  **Control (n=351)**  Endline 24.80±4.94  **Group-only (n=346)**  Endline 25.69±5.42  **Mixed-delivery (n=373**)  Endline 25.77±5.19 |
| Yousafzai *et al.* (2014)^9^; Pakistan | **BSID-III cognitive scale composite score**  **Responsive stimulation intervention (n=696 at 12 mo, n=701 at 24 mo)**  12mo 97.1 ±14.2/ 24mo 81.7 ±14.7  **No responsive stimulation intervention (n=661 at 12 mo, n=680 at 24 mo)**  12mo 92.0 ±13.0 / 24mo 74.1 ±13.5  **Enhanced nutrition intervention**  **(n=658 at 12 mo, n=676 at 24 mo)**  12mo 95.9±12.7 / 24mo 78.4 ±14.6  **No Enhanced nutrition intervention (n=699 at 12 mo, n= 705 at 24 mo)**  12mo 93.4 ±14.8 / 24mo 77.6 ±14**.**7 | **Bayley III Motor scale**  **Responsive stimulation intervention (n=696 at 12 mo, n=701 at 24 mo)**  12mo 83.7 ±14.3 / 24mo 92.1±17.3  **No responsive stimulation intervention (n=661 at 12 mo, n=680 at 24 mo)**  12mo 80.81 ±2.9 / 24mo 84.8±16.4  **Enhanced nutrition intervention**  **(n=658 at 12 mo, n=676 at 24 mo)**  12mo 83.2 ±13.4 / 24mo 89.4±16.8  **No enhanced nutrition intervention (n=699 at 12 mo, n= 705 at 24 mo)**  12mo 81.5 ±13.9 / 24mo 87.7±17.5 | **Bayley III**  **Responsive stimulation intervention (n=696 at 12 mo, n=701 at 24 mo)**  12mo 77.1±14.8/ 24mo 85.7±13.3  **No responsive stimulation intervention (n=661 at 12 mo, n=680 at 24 mo)**  12mo 72.5±12.5/ 24mo 79.3±12.8  **Enhanced nutrition intervention**  **(n=658 at 12 mo, n=676 at 24 mo)**  12mo 76.8±12.9/ 24mo 84.3±13.7  **No enhanced nutrition intervention (n=699 at 12 mo, n= 705 at 24 mo)**  12mo 73.0±14.7 / 24mo 80.9±12.9 | **Bayley III**  **Responsive stimulation intervention (n=696 at 12 mo, n=701 at 24 mo)**  12mo 80.5±13.4/ 24mo 93.2±18.4;  **No responsive stimulation intervention (n=661 at 12 mo, n=680 at 24 mo)**  12mo 77.6±13.8/ 24mo 94.9±39.3  **Enhanced nutrition intervention**  **(n=658 at 12 mo, n=676 at 24 mo)**  12mo 81.0±13.7/ 24mo 94.8±20.3  **No enhanced nutrition intervention (n=699 at 12 mo, n= 705 at 24 mo)**  12mo 77.2±13.5/ 24mo 93.3±37.8 |
| Caridad Araujov *et al.* (2021)^10^ ; Peru | ASQ-3 (Cognitive development)  Not reported | ASQ-3 (Fine motor & Gross motor)  Not reported | ASQ-3 (communication)  Not reported | ASQ-3 (Personal social)  Not reported |

^1^ Hamadani JD, Huda SN, Khatun F, Grantham-mcgregor SM. Psychosocial stimulation improves the development of undernourished children in rural Bangladesh 1. J Nutr Ingestive Behav Neurosci. 2006; 2645–2652.

^2^ Eickmann SH, Lima AC V, Guerra MQ, Lima MC, Lira PIC, Huttly SRA, et al. Improved cognitive and motor development in a community-based intervention of psychosocial stimulation in northeast Brazil. Dev Med Child Neurol. 2003;45: 536–541. doi:10.1017/s0012162203000987.

^3^ Attanasio OP, Fernández C, Fitzsimons EOA, Grantham-mcgregor SM, Meghir C, Rubio-codina M. Using the infrastructure of a conditional cash transfer program to deliver a scalable integrated early child development program in Colombia : cluster randomized controlled trial. BMJ. 2014;349: g5785. doi:10.1136/bmj.g5785.

^4^ Grantham-McGregor S, Adya A, Attanasio O, Augsburg B, Behrman J, Caeyers B, et al. Group sessions or home visits for early childhood development in India: a cluster RCT. Pediatrics. 2020;146. doi: 10.1542/peds.2020-002725.

^5^ Gardner JMM, Powell CA, Baker-Henningham H, Walker SP, Cole TJ, Grantham-McGregor SM. Zinc supplementation and psychosocial stimulation: effects on the development of undernourished Jamaican children–. Am J Clin Nutr. 2005;82(2):399–405.

^6^ Powell C, Baker-Henningham H, Walker S, Gernay J, Grantham-McGregor S. Feasibility of integrating early stimulation into primary care for undernourished Jamaican children: cluster randomised controlled trial. BMJ. 2004;329: 89.

^7^ Lopez Garcia I, Saya UY, Luoto JE. Cost-effectiveness and economic returns of group-based parenting interventions to promote early childhood development: Results from a randomized controlled trial in rural Kenya. PLOS Med. 2021;18: e1003746. doi:10.1371/journal.pmed.1003746.

^8^ Luoto JE, Garcia IL, Aboud FE, Singla DR, Fernald LCH, Pitchik HO, et al. Group-based parenting interventions to promote child development in rural Kenya: a multi-arm, cluster-randomised community effectiveness trial. Lancet Glob Heal. 2021;9: e309–e319.

^9^ Yousafzai AK, Rasheed MA, Siyal S. Integration of parenting and nutrition interventions in a community health program in Pakistan:an implementation evaluation. Ann N Y Acad Sci. 2018;1419: 160–178. doi:10.1111/nyas.13649.

^10^ Caridad Araujov M, Dormal M, Grantham-McGregor S, Lazarte F, Rubio-Codina M, Schady N. Home visiting at scale and child development. J Public Econ Plus. 2021;2: 100003. doi: 10.1016/j.pubecp.2021.100003.
