# Supplementary material for: Measuring the cost-effectiveness of a home-visiting intervention to promote early child development among rural families linked to the Rwandan social protection system
Source: PLOS Glob Public Health. 2023 Oct 24;3(10):e0002473. doi: 10.1371/journal.pgph.0002473 (PMC10597512; doi:10.1371/journal.pgph.0002473)
Supplement: S3 Table — (DOCX) [file pgph.0002473.s003.docx]

**S3 Table. Selected interventions reported standardised impact on development**

| **Study** | **Intervention** | **Country** | **No. of home visits/group sessions (intended)** | **Treatment effect (Standard deviation)** | | | |
| --- | --- | --- | --- | --- | --- | --- | --- |
|  |  |  |  | **Cognitive** | **Language** | **Motor** | **Socio-emotional** |
|  | Sugira Muryango | Rwanda | 12/0 | 0.11 | 0.081 | 0.162 | 0.096 |
| Hamadani *et al.* (2006)^1^ | Centre based nutrition supplementation + psychosocial stimulation | Bangladesh | 80/44 | 0.33 |  | 0.16^b^ | 0.21 ^b^ |
| Eickmann *et al.* (2003)^2^ | Psychosocial stimulation | Brazil | 11/3 | 0.5 |  | 0.5 |  |
| Attanasio *et al.* (2014)^3^ | Psychosocial stimulation | Colombia | 78/0 | 0.260 | 0.22^c^ | 0.122 |  |
|  |  |  |  |  | 0.084^d^ |  |  |
| Grantham-McGregor (2020)^4^ | Psychosocial stimulation + nutritional education | India | 96/0 | 0.324 | 0.239 | 0.055 |  |
| Gardner et al., **(**2005)^5^ | Psychosocial stimulation | Jamaica | 24/0 | 0.22 ^b^ | 0.26 ^b^ | -0.36 ^b^ |  |
| Powell et al., (2004)^6^ | Psychosocial stimulation | Jamaica | 50/0 | 0.86^a^ | 0.77 ^b^ | 0.39^b^ |  |
| Lopez Garcia *et al.* (2021)^7^ &  Luoto *et al.* (2021)^8^ | Psychosocial stimulation + nutrition education | Kenya | 4/12 | 0.34 | 0.20 ^c^  -0.09^d^ |  | 0.22 |
| Yousafzai *et al.* (2014)^9^ | Psychosocial stimulation | Pakistan | 24/24 | 0.6 | 0.7 | 0.5 |  |
| Caridad Araujov *et al.* (2021)^10^ | Psychosocial stimulation | Peru | 10/0 | 0.022^e^ | 0.025^e^ | 0.016^f^  -0.005^g^ | 0.016 |

^a^ (Jensen et al., 2021)^11^

^b^ (Zhang et al., 2022)^12^

^c^ Receptive language

^d^ Expressive language

^e^ Treatment on the treated

^f^ Fine motor

^g^ Gross motor

^1^ Hamadani JD, Huda SN, Khatun F, Grantham-mcgregor SM. Psychosocial stimulation improves the development of undernourished children in rural Bangladesh 1. J Nutr Ingestive Behav Neurosci. 2006; 2645–2652.

^2^ Eickmann SH, Lima AC V, Guerra MQ, Lima MC, Lira PIC, Huttly SRA, et al. Improved cognitive and motor development in a community-based intervention of psychosocial stimulation in northeast Brazil. Dev Med Child Neurol. 2003;45: 536–541. doi:10.1017/s0012162203000987.

^3^ Attanasio OP, Fernández C, Fitzsimons EOA, Grantham-mcgregor SM, Meghir C, Rubio-codina M. Using the infrastructure of a conditional cash transfer program to deliver a scalable integrated early child development program in Colombia : cluster randomized controlled trial. BMJ. 2014;349: g5785. doi:10.1136/bmj.g5785.

^4^ Grantham-McGregor S, Adya A, Attanasio O, Augsburg B, Behrman J, Caeyers B, et al. Group sessions or home visits for early childhood development in India: a cluster RCT. Pediatrics. 2020;146. doi: 10.1542/peds.2020-002725.

^5^ Gardner JMM, Powell CA, Baker-Henningham H, Walker SP, Cole TJ, Grantham-McGregor SM. Zinc supplementation and psychosocial stimulation: effects on the development of undernourished Jamaican children–. Am J Clin Nutr. 2005;82(2):399–405.

^6^ Powell C, Baker-Henningham H, Walker S, Gernay J, Grantham-McGregor S. Feasibility of integrating early stimulation into primary care for undernourished Jamaican children: cluster randomised controlled trial. BMJ. 2004;329: 89.

^7^ Lopez Garcia I, Saya UY, Luoto JE. Cost-effectiveness and economic returns of group-based parenting interventions to promote early childhood development: Results from a randomized controlled trial in rural Kenya. PLOS Med. 2021;18: e1003746. doi:10.1371/journal.pmed.1003746.

^8^ Luoto JE, Garcia IL, Aboud FE, Singla DR, Fernald LCH, Pitchik HO, et al. Group-based parenting interventions to promote child development in rural Kenya: a multi-arm, cluster-randomised community effectiveness trial. Lancet Glob Heal. 2021;9: e309–e319.

^9^ Yousafzai AK, Rasheed MA, Siyal S. Integration of parenting and nutrition interventions in a community health program in Pakistan:an implementation evaluation. Ann N Y Acad Sci. 2018;1419: 160–178. doi:10.1111/nyas.13649.

^10^ Caridad Araujov M, Dormal M, Grantham-McGregor S, Lazarte F, Rubio-Codina M, Schady N. Home visiting at scale and child development. J Public Econ Plus. 2021;2: 100003. doi: 10.1016/j.pubecp.2021.100003.

^11 J^ensen SK, Placencio-Castro M, Murray SM, Brennan RT, Goshev S, Farrar J, et al. Effect of a home-visiting parenting program to promote early childhood development and prevent violence: a cluster-randomized trial in Rwanda. BMJ Glob Heal. 2021;6. doi:10.1136/bmjgh-2020-003508

^12^ Zhang L, Ssewanyana D, Martin M-C, Lye S, Moran G, Abubakar A, et al. Supporting child development through parenting interventions in low-to middle-income countries: an updated systematic review. Front public Heal. 2021;9.

**S4 Table. Assumptions applied to expenditure data**

| Assumption | **Value** |
| --- | --- |
| Managers’ use of transport relative to supervisors | 0.5 |
| Project director use of transport relative to supervisors | 0.5 |
| Allocation of hosting NGO overheads allocated to implementation | 0.5 |
| Discount rate (the most commonly used in studies in the region) | 3%, |
| Exchange rate to US$ | 900 RWF |

**S5 Table. Capital costs average useful life**

| **Assets** | **Cost USD** | **Average useful life** | **Rationale** | **Annual value USD** |
| --- | --- | --- | --- | --- |
| Recorders and Batteries for CBVs | 2030 | 2 years | Rapid advancements in technology and wear and tear | 1061 |
| Laptops for reporting | 2871 | 2 years |  | 1500 |
| Sim-cards and modems | 2600 | 2 years |  | 1300 |

**S6 Table. Costing inputs**

| **Parameter** | | **Data (year)** | **Source** | **Notes** | **Final input** |
| --- | --- | --- | --- | --- | --- |
| Wage rate, no schooling | Vision 2020 Umurenge Programme (VUP) benefits | Direct support: up to RWF21,000 monthly  Public works: up to RWF1,500 per workday | Social Protection.Org^1^ | Direct support: Households are eligible if they are poor and labour constrained.  Public works: Households are only eligible if they are both extremely poor and with able-bodied members. | $21 per month |
| Returns to education per year | Return to another year of schooling (% increase in earnings) | 14.7; SD 3.9 (1997)  17.5; SD 4.5 (2005)  22.4; SD 3.8 (2010) | Montenegro & Patrinos (2014)^2^ |  | 22.4% |
| Years of schooling, under 25 years | Median years completed | \| Age \| Median years completed \| \|  \| \| --- \| --- \| --- \| --- \| \| **Male** \| **Female** \| **Average M & F** \| \| **6-9** \| 0.3 \| 0.5 \| 0.4 \| \| **10-14** \| 3.1 \| 3.6 \| 3.35 \| \| **15-19** \| 5.4 \| 5.7 \| 5.55 \| \| **20-24** \| 5.5 \| 5.9 \| 5.7 \| | National Institute of Statistics of Rwanda^3^ |  | 6 years |
| GDP per capita growth/ wage growth | GDP per capita growth (annual %) | 3.25 (2016)  1.27(2017)  5.75 (2018)  6.64 (2019)  -5.783 (2020)  2.23 (average 2016-2020) | World Bank national accounts data, and OECD National Accounts data files^.4^ | “Annual percentage growth rate of GDP per capita based on constant local currency. Aggregates are based on constant 2010 U.S. dollars.  GDP per capita is gross domestic product divided by midyear population. GDP at purchaser's prices is the sum of gross value added by all resident producers in the economy plus any product taxes and minus any subsidies not included in the value of the products. It is calculated without making deductions for depreciation of fabricated assets or for depletion and degradation of natural resources.”^4^ | 5% |
| Employment rate | Employment to population ratio, 15+, total (%) (modeled ILO estimate) | 82,83 (2018)  82,73 (2019)  79,24 (2020) | International Labour Organization, ILOSTAT database. Data retrieved on June 15, 2021.^5^ | “Employment to population ratio is the proportion of a country's population that is employed. Employment is defined as persons of working age who, during a short reference period, were engaged in any activity to produce goods or provide services for pay or profit, whether at work during the reference period (i.e. who worked in a job for at least one hour) or not at work due to temporary absence from a job, or to working-time arrangements. Ages 15 and older are generally considered the working-age population.”^5^ | 80% |
| Years of working life | Years of working life | Assumption | Assumption |  | 40 years |
| Years until entry into labour market | Years until entry into labour market | Assumption | Assumption |  | 16 years |
| Impact on schooling | Years of schooling | 0.07 – 0.11 | Engle et al. (2007)^6^ | Upper and lower estimates reported by Engle et al^6^ scaled to the effect size for SM |  |

^1^ Social protection.org. (16 November 2021). Vision 2020 Umurenge Programme (VUP). Available: https://socialprotection.org/discover/programmes/vision-2020-umurenge-programme-vup. (Accessed 17 November 2021).

^2^ Montenegro CE, & Patrinos HA. Comparable estimates of returns to schooling around the world (September 1, 2014).; World Bank Policy Research Working Paper No. 7020. Available: <https://ssrn.com/abstract=2491933>. (Accessed 17 November 2021).

^3^ National Institute of Statistics of Rwanda (NISR) [Rwanda], Ministry of Health (MOH) [Rwanda], and ICF. 2021. Rwanda Demographic and Health Survey 2019-20 Final Report. Kigali, Rwanda, and Rockville, Maryland, USA: NISR and ICF. Available: <https://dhsprogram.com/publications/publication-FR370-DHS-Final-Reports.cfm> (Accessed 17 November 2021).

^4^ World Bank national accounts data, and OECD National Accounts data files. Available: https://data.worldbank.org/indicator/NY.GDP.PCAP.KD.ZG?locations=RW. (Accessed 17 November 2021).

^5^ International Labour Organization, ILOSTAT database. Data retrieved on June 15, 2021.Available: <https://data.worldbank.org/country/rwanda>. (Accessed 17 November 2021).

^6^ Engle PL, Black MM, Behrman JR, De Mello MC, Gertler PJ, Kapiriri L, Martorell R, Young ME, & International Child Development Steering Group. (2007). Strategies to avoid the loss of developmental potential in more than 200 million children in the developing world. Lancet. 2007;369: 229–242.

**S7 Table. Consolidated Health Economic Evaluation Reporting Standards 2022 (CHEERS 2022) Checklist^1^**

| **Topic** | **No.** | **Item** | **Location where item is reported** |
| --- | --- | --- | --- |
| **Title** | 1 | Identify the study as an economic evaluation and specify the interventions being compared. | Title, page 1 |
| **Abstract** | 2 | Provide a structured summary that highlights context, key methods, results, and alternative analyses. | Abstract, page 2 |
| **Introduction** | | | |
| **Background and objectives** | 3 | Give the context for the study, the study question, and its practical relevance for decision making in policy or practice. | Introduction, page 2-4.  The Sugira Muryango Trial, pages 4-5 |
| **Methods** | | | |
| **Health economic analysis plan** | 4 | Indicate whether a health economic analysis plan was developed and where available. | None. |
| **Study population** | 5 | Describe characteristics of the study population (such as age range, demographics, socioeconomic, or clinical characteristics). | The Sugira Muryango Trial, pagea 4-5 |
| **Setting and location** | 6 | Provide relevant contextual information that may influence findings. | The Sugira Muryango Trial, page 4-5 |
| **Comparators** | 7 | Describe the interventions or strategies being compared and why chosen. | Cost-effectiveness analysis pages 8-9&  Supplementary material, S1 Table Comparison of interventions with a home-visiting and psychosocial stimulation component with positive early cognitive outcomes |
| **Perspective** | 8 | State the perspective(s) adopted by the study and why chosen. | Methods page 4-9 |
| **Time horizon** | 9 | State the time horizon for the study and why appropriate. | Supplementary Material, S4 Table. Assumptions applied to expenditure data |
| **Discount rate** | 10 | Report the discount rate(s) and reason chosen. | Supplementary Material, S4 Table. Assumptions applied to expenditure data |
| **Selection of outcomes** | 11 | Describe what outcomes were used as the measure(s) of benefit(s) and harm(s). | Cost-effectiveness analysis, pages 8-9 |
| **Measurement of outcomes** | 12 | Describe how outcomes used to capture benefit(s) and harm(s) were measured. | Supplementary material, S1 Table. Comparison of interventions with a home-visiting and psychosocial stimulation component with positive early cognitive outcomes |
| **Valuation of outcomes** | 13 | Describe the population and methods used to measure and value outcomes. | Supplementary material, S1 Table. Comparison of interventions with a home-visiting and psychosocial stimulation component with positive early cognitive outcomes |
| **Measurement and valuation of resources and costs** | 14 | Describe how costs were valued. | Cost-analysis of 3 scenarios, pages 5-6  &  Table 2. Intervention implementation scenarios  &  Supplementary Material, S6 Table. Costing inputs |
| **Currency, price date, and conversion** | 15 | Report the dates of the estimated resource quantities and unit costs, plus the currency and year of conversion. | Supplementary material, S4 Table. Assumptions applied to expenditure data |
| **Rationale and description of model** | 16 | If modelling is used, describe in detail and why used. Report if the model is publicly available and where it can be accessed. | Methods, Page 4-9 |
| **Analytics and assumptions** | 17 | Describe any methods for analysing or statistically transforming data, any extrapolation methods, and approaches for validating any model used. | N/A |
| **Characterising heterogeneity** | 18 | Describe any methods used for estimating how the results of the study vary for subgroups. | N/A |
| **Characterising distributional effects** | 19 | Describe how impacts are distributed across different individuals or adjustments made to reflect priority populations. | N/A |
| **Characterising uncertainty** | 20 | Describe methods to characterise any sources of uncertainty in the analysis. | N/A |
| **Approach to engagement with patients and others affected by the study** | 21 | Describe any approaches to engage patients or service recipients, the general public, communities, or stakeholders (such as clinicians or payers) in the design of the study. | None |
| **Results** | | | |
| **Study parameters** | 22 | Report all analytic inputs (such as values, ranges, references) including uncertainty or distributional assumptions. | Supplementary material, S4 Table. Assumptions applied to expenditure data |
| **Summary of main results** | 23 | Report the mean values for the main categories of costs and outcomes of interest and summarise them in the most appropriate overall measure. | Results, pages 9-10 |
| **Effect of uncertainty** | 24 | Describe how uncertainty about analytic judgments, inputs, or projections affect findings. Report the effect of choice of discount rate and time horizon, if applicable. | Methods, Page 4-9 |
| **Effect of engagement with patients and others affected by the study** | 25 | Report on any difference patient/service recipient, general public, community, or stakeholder involvement made to the approach or findings of the study | None |
| **Discussion** | | | |
| **Study findings, limitations, generalisability, and current knowledge** | 26 | Report key findings, limitations, ethical or equity considerations not captured, and how these could affect patients, policy, or practice. | Discussion, pages 12-14 |
| **Other relevant information** | | | |
| **Source of funding** | 27 | Describe how the study was funded and any role of the funder in the identification, design, conduct, and reporting of the analysis | Funding, page TBD |
| **Conflicts of interest** | 28 | Report authors conflicts of interest according to journal or International Committee of Medical Journal Editors requirements. | Conflicts of interest/Competing interests, page TBD |

^1^ Husereau D, Drummond M, Augustovski F, et al. Consolidated Health Economic Evaluation Reporting Standards 2022 (CHEERS 2022) Explanation and Elaboration: A Report of the ISPOR CHEERS II Good Practices Task Force. Value Health 2022;25. doi:10.1016/j.jval.2021.10.008
