# Supplementary material for: Measuring the cost-effectiveness of a home-visiting intervention to promote early child development among rural families linked to the Rwandan social protection system
Source: PLOS Glob Public Health. 2023 Oct 24;3(10):e0002473. doi: 10.1371/journal.pgph.0002473 (PMC10597512; doi:10.1371/journal.pgph.0002473)
Supplement: S4 Table — (DOCX) [file pgph.0002473.s004.docx]

**S4 Table. Assumptions applied to expenditure data**

| Assumption | **Value** |
| --- | --- |
| Managers’ use of transport relative to supervisors | 0.5 |
| Project director use of transport relative to supervisors | 0.5 |
| Allocation of hosting NGO overheads allocated to implementation | 0.5 |
| Discount rate (the most commonly used in studies in the region) | 3%, |
| Exchange rate to US$ | 900 RWF |
