# Supplementary material for: Measuring the cost-effectiveness of a home-visiting intervention to promote early child development among rural families linked to the Rwandan social protection system
Source: PLOS Glob Public Health. 2023 Oct 24;3(10):e0002473. doi: 10.1371/journal.pgph.0002473 (PMC10597512; doi:10.1371/journal.pgph.0002473)
Supplement: S5 Table — (DOCX) [file pgph.0002473.s005.docx]

**S5 Table. Capital costs average useful life**

| **Assets** | **Cost USD** | **Average useful life** | **Rationale** | **Annual value USD** |
| --- | --- | --- | --- | --- |
| Recorders and Batteries for CBVs | 2030 | 2 years | Rapid advancements in technology and wear and tear | 1061 |
| Laptops for reporting | 2871 | 2 years |  | 1500 |
| Sim-cards and modems | 2600 | 2 years |  | 1300 |
