# Supplementary material for: Measuring the cost-effectiveness of a home-visiting intervention to promote early child development among rural families linked to the Rwandan social protection system
Source: PLOS Glob Public Health. 2023 Oct 24;3(10):e0002473. doi: 10.1371/journal.pgph.0002473 (PMC10597512; doi:10.1371/journal.pgph.0002473)
Supplement: S6 Table — (DOCX) [file pgph.0002473.s006.docx]

**S6 Table. Costing inputs**

| **Parameter** | | **Data (year)** | **Source** | **Notes** | **Final input** |
| --- | --- | --- | --- | --- | --- |
| Wage rate, no schooling | Vision 2020 Umurenge Programme (VUP) benefits | Direct support: up to RWF21,000 monthly  Public works: up to RWF1,500 per workday | Social Protection.Org^1^ | Direct support: Households are eligible if they are poor and labour constrained.  Public works: Households are only eligible if they are both extremely poor and with able-bodied members. | $21 per month |
| Returns to education per year | Return to another year of schooling (% increase in earnings) | 14.7; SD 3.9 (1997)  17.5; SD 4.5 (2005)  22.4; SD 3.8 (2010) | Montenegro & Patrinos (2014)^2^ |  | 22.4% |
| Years of schooling, under 25 years | Median years completed | \| Age \| Median years completed \| \|  \| \| --- \| --- \| --- \| --- \| \| **Male** \| **Female** \| **Average M & F** \| \| **6-9** \| 0.3 \| 0.5 \| 0.4 \| \| **10-14** \| 3.1 \| 3.6 \| 3.35 \| \| **15-19** \| 5.4 \| 5.7 \| 5.55 \| \| **20-24** \| 5.5 \| 5.9 \| 5.7 \| | National Institute of Statistics of Rwanda^3^ |  | 6 years |
| GDP per capita growth/ wage growth | GDP per capita growth (annual %) | 3.25 (2016)  1.27(2017)  5.75 (2018)  6.64 (2019)  -5.783 (2020)  2.23 (average 2016-2020) | World Bank national accounts data, and OECD National Accounts data files^.4^ | “Annual percentage growth rate of GDP per capita based on constant local currency. Aggregates are based on constant 2010 U.S. dollars.  GDP per capita is gross domestic product divided by midyear population. GDP at purchaser's prices is the sum of gross value added by all resident producers in the economy plus any product taxes and minus any subsidies not included in the value of the products. It is calculated without making deductions for depreciation of fabricated assets or for depletion and degradation of natural resources.”^4^ | 5% |
| Employment rate | Employment to population ratio, 15+, total (%) (modeled ILO estimate) | 82,83 (2018)  82,73 (2019)  79,24 (2020) | International Labour Organization, ILOSTAT database. Data retrieved on June 15, 2021.^5^ | “Employment to population ratio is the proportion of a country's population that is employed. Employment is defined as persons of working age who, during a short reference period, were engaged in any activity to produce goods or provide services for pay or profit, whether at work during the reference period (i.e. who worked in a job for at least one hour) or not at work due to temporary absence from a job, or to working-time arrangements. Ages 15 and older are generally considered the working-age population.”^5^ | 80% |
| Years of working life | Years of working life | Assumption | Assumption |  | 40 years |
| Years until entry into labour market | Years until entry into labour market | Assumption | Assumption |  | 16 years |
| Impact on schooling | Years of schooling | 0.07 – 0.11 | Engle et al. (2007)^6^ | Upper and lower estimates reported by Engle et al^6^ scaled to the effect size for SM |  |

^1^ Social protection.org. (16 November 2021). Vision 2020 Umurenge Programme (VUP). Available: https://socialprotection.org/discover/programmes/vision-2020-umurenge-programme-vup. (Accessed 17 November 2021).

^2^ Montenegro CE, & Patrinos HA. Comparable estimates of returns to schooling around the world (September 1, 2014).; World Bank Policy Research Working Paper No. 7020. Available: <https://ssrn.com/abstract=2491933>. (Accessed 17 November 2021).

^3^ National Institute of Statistics of Rwanda (NISR) [Rwanda], Ministry of Health (MOH) [Rwanda], and ICF. 2021. Rwanda Demographic and Health Survey 2019-20 Final Report. Kigali, Rwanda, and Rockville, Maryland, USA: NISR and ICF. Available: <https://dhsprogram.com/publications/publication-FR370-DHS-Final-Reports.cfm> (Accessed 17 November 2021).

^4^ World Bank national accounts data, and OECD National Accounts data files. Available: https://data.worldbank.org/indicator/NY.GDP.PCAP.KD.ZG?locations=RW. (Accessed 17 November 2021).

^5^ International Labour Organization, ILOSTAT database. Data retrieved on June 15, 2021.Available: <https://data.worldbank.org/country/rwanda>. (Accessed 17 November 2021).

^6^ Engle PL, Black MM, Behrman JR, De Mello MC, Gertler PJ, Kapiriri L, Martorell R, Young ME, & International Child Development Steering Group. (2007). Strategies to avoid the loss of developmental potential in more than 200 million children in the developing world. Lancet. 2007;369: 229–242.
