# Supplementary material for: Measuring the cost-effectiveness of a home-visiting intervention to promote early child development among rural families linked to the Rwandan social protection system
Source: PLOS Glob Public Health. 2023 Oct 24;3(10):e0002473. doi: 10.1371/journal.pgph.0002473 (PMC10597512; doi:10.1371/journal.pgph.0002473)
Supplement: S7 Table — (DOCX) [file pgph.0002473.s007.docx]

**S3 Table. Selected interventions reported standardised impact on development**

| **Study** | **Intervention** | **Country** | **No. of home visits/group sessions (intended)** | **Treatment effect (Standard deviation)** | | | |
| --- | --- | --- | --- | --- | --- | --- | --- |
|  |  |  |  | **Cognitive** | **Language** | **Motor** | **Socio-emotional** |
|  | Sugira Muryango | Rwanda | 12/0 | 0.11 | 0.081 | 0.162 | 0.096 |
| Hamadani *et al.* (2006)^1^ | Centre based nutrition supplementation + psychosocial stimulation | Bangladesh | 80/44 | 0.33 |  | 0.16^b^ | 0.21 ^b^ |
| Eickmann *et al.* (2003)^2^ | Psychosocial stimulation | Brazil | 11/3 | 0.5 |  | 0.5 |  |
| Attanasio *et al.* (2014)^3^ | Psychosocial stimulation | Colombia | 78/0 | 0.260 | 0.22^c^ | 0.122 |  |
|  |  |  |  |  | 0.084^d^ |  |  |
| Grantham-McGregor (2020)^4^ | Psychosocial stimulation + nutritional education | India | 96/0 | 0.324 | 0.239 | 0.055 |  |
| Gardner et al., **(**2005)^5^ | Psychosocial stimulation | Jamaica | 24/0 | 0.22 ^b^ | 0.26 ^b^ | -0.36 ^b^ |  |
| Powell et al., (2004)^6^ | Psychosocial stimulation | Jamaica | 50/0 | 0.86^a^ | 0.77 ^b^ | 0.39^b^ |  |
| Lopez Garcia *et al.* (2021)^7^ &  Luoto *et al.* (2021)^8^ | Psychosocial stimulation + nutrition education | Kenya | 4/12 | 0.34 | 0.20 ^c^  -0.09^d^ |  | 0.22 |
| Yousafzai *et al.* (2014)^9^ | Psychosocial stimulation | Pakistan | 24/24 | 0.6 | 0.7 | 0.5 |  |
| Caridad Araujov *et al.* (2021)^10^ | Psychosocial stimulation | Peru | 10/0 | 0.022^e^ | 0.025^e^ | 0.016^f^  -0.005^g^ | 0.016 |

^a^ (Jensen et al., 2021)^11^

^b^ (Zhang et al., 2022)^12^

^c^ Receptive language

^d^ Expressive language

^e^ Treatment on the treated

^f^ Fine motor

^g^ Gross motor

^1^ Hamadani JD, Huda SN, Khatun F, Grantham-mcgregor SM. Psychosocial stimulation improves the development of undernourished children in rural Bangladesh 1. J Nutr Ingestive Behav Neurosci. 2006; 2645–2652.

^2^ Eickmann SH, Lima AC V, Guerra MQ, Lima MC, Lira PIC, Huttly SRA, et al. Improved cognitive and motor development in a community-based intervention of psychosocial stimulation in northeast Brazil. Dev Med Child Neurol. 2003;45: 536–541. doi:10.1017/s0012162203000987.

^3^ Attanasio OP, Fernández C, Fitzsimons EOA, Grantham-mcgregor SM, Meghir C, Rubio-codina M. Using the infrastructure of a conditional cash transfer program to deliver a scalable integrated early child development program in Colombia : cluster randomized controlled trial. BMJ. 2014;349: g5785. doi:10.1136/bmj.g5785.

^4^ Grantham-McGregor S, Adya A, Attanasio O, Augsburg B, Behrman J, Caeyers B, et al. Group sessions or home visits for early childhood development in India: a cluster RCT. Pediatrics. 2020;146. doi: 10.1542/peds.2020-002725.

^5^ Gardner JMM, Powell CA, Baker-Henningham H, Walker SP, Cole TJ, Grantham-McGregor SM. Zinc supplementation and psychosocial stimulation: effects on the development of undernourished Jamaican children–. Am J Clin Nutr. 2005;82(2):399–405.

^6^ Powell C, Baker-Henningham H, Walker S, Gernay J, Grantham-McGregor S. Feasibility of integrating early stimulation into primary care for undernourished Jamaican children: cluster randomised controlled trial. BMJ. 2004;329: 89.

^7^ Lopez Garcia I, Saya UY, Luoto JE. Cost-effectiveness and economic returns of group-based parenting interventions to promote early childhood development: Results from a randomized controlled trial in rural Kenya. PLOS Med. 2021;18: e1003746. doi:10.1371/journal.pmed.1003746.

^8^ Luoto JE, Garcia IL, Aboud FE, Singla DR, Fernald LCH, Pitchik HO, et al. Group-based parenting interventions to promote child development in rural Kenya: a multi-arm, cluster-randomised community effectiveness trial. Lancet Glob Heal. 2021;9: e309–e319.

^9^ Yousafzai AK, Rasheed MA, Siyal S. Integration of parenting and nutrition interventions in a community health program in Pakistan:an implementation evaluation. Ann N Y Acad Sci. 2018;1419: 160–178. doi:10.1111/nyas.13649.

^10^ Caridad Araujov M, Dormal M, Grantham-McGregor S, Lazarte F, Rubio-Codina M, Schady N. Home visiting at scale and child development. J Public Econ Plus. 2021;2: 100003. doi: 10.1016/j.pubecp.2021.100003.

^11 J^ensen SK, Placencio-Castro M, Murray SM, Brennan RT, Goshev S, Farrar J, et al. Effect of a home-visiting parenting program to promote early childhood development and prevent violence: a cluster-randomized trial in Rwanda. BMJ Glob Heal. 2021;6. doi:10.1136/bmjgh-2020-003508

^12^ Zhang L, Ssewanyana D, Martin M-C, Lye S, Moran G, Abubakar A, et al. Supporting child development through parenting interventions in low-to middle-income countries: an updated systematic review. Front public Heal. 2021;9.

**S7 Table. Consolidated Health Economic Evaluation Reporting Standards 2022 (CHEERS 2022) Checklist^1^**

| **Topic** | **No.** | **Item** | **Location where item is reported** |
| --- | --- | --- | --- |
| **Title** | 1 | Identify the study as an economic evaluation and specify the interventions being compared. | Title, page 1 |
| **Abstract** | 2 | Provide a structured summary that highlights context, key methods, results, and alternative analyses. | Abstract, page 2 |
| **Introduction** | | | |
| **Background and objectives** | 3 | Give the context for the study, the study question, and its practical relevance for decision making in policy or practice. | Introduction, page 2-4.  The Sugira Muryango Trial, pages 4-5 |
| **Methods** | | | |
| **Health economic analysis plan** | 4 | Indicate whether a health economic analysis plan was developed and where available. | None. |
| **Study population** | 5 | Describe characteristics of the study population (such as age range, demographics, socioeconomic, or clinical characteristics). | The Sugira Muryango Trial, pagea 4-5 |
| **Setting and location** | 6 | Provide relevant contextual information that may influence findings. | The Sugira Muryango Trial, page 4-5 |
| **Comparators** | 7 | Describe the interventions or strategies being compared and why chosen. | Cost-effectiveness analysis pages 8-9&  Supplementary material, S1 Table Comparison of interventions with a home-visiting and psychosocial stimulation component with positive early cognitive outcomes |
| **Perspective** | 8 | State the perspective(s) adopted by the study and why chosen. | Methods page 4-9 |
| **Time horizon** | 9 | State the time horizon for the study and why appropriate. | Supplementary Material, S4 Table. Assumptions applied to expenditure data |
| **Discount rate** | 10 | Report the discount rate(s) and reason chosen. | Supplementary Material, S4 Table. Assumptions applied to expenditure data |
| **Selection of outcomes** | 11 | Describe what outcomes were used as the measure(s) of benefit(s) and harm(s). | Cost-effectiveness analysis, pages 8-9 |
| **Measurement of outcomes** | 12 | Describe how outcomes used to capture benefit(s) and harm(s) were measured. | Supplementary material, S1 Table. Comparison of interventions with a home-visiting and psychosocial stimulation component with positive early cognitive outcomes |
| **Valuation of outcomes** | 13 | Describe the population and methods used to measure and value outcomes. | Supplementary material, S1 Table. Comparison of interventions with a home-visiting and psychosocial stimulation component with positive early cognitive outcomes |
| **Measurement and valuation of resources and costs** | 14 | Describe how costs were valued. | Cost-analysis of 3 scenarios, pages 5-6  &  Table 2. Intervention implementation scenarios  &  Supplementary Material, S6 Table. Costing inputs |
| **Currency, price date, and conversion** | 15 | Report the dates of the estimated resource quantities and unit costs, plus the currency and year of conversion. | Supplementary material, S4 Table. Assumptions applied to expenditure data |
| **Rationale and description of model** | 16 | If modelling is used, describe in detail and why used. Report if the model is publicly available and where it can be accessed. | Methods, Page 4-9 |
| **Analytics and assumptions** | 17 | Describe any methods for analysing or statistically transforming data, any extrapolation methods, and approaches for validating any model used. | N/A |
| **Characterising heterogeneity** | 18 | Describe any methods used for estimating how the results of the study vary for subgroups. | N/A |
| **Characterising distributional effects** | 19 | Describe how impacts are distributed across different individuals or adjustments made to reflect priority populations. | N/A |
| **Characterising uncertainty** | 20 | Describe methods to characterise any sources of uncertainty in the analysis. | N/A |
| **Approach to engagement with patients and others affected by the study** | 21 | Describe any approaches to engage patients or service recipients, the general public, communities, or stakeholders (such as clinicians or payers) in the design of the study. | None |
| **Results** | | | |
| **Study parameters** | 22 | Report all analytic inputs (such as values, ranges, references) including uncertainty or distributional assumptions. | Supplementary material, S4 Table. Assumptions applied to expenditure data |
| **Summary of main results** | 23 | Report the mean values for the main categories of costs and outcomes of interest and summarise them in the most appropriate overall measure. | Results, pages 9-10 |
| **Effect of uncertainty** | 24 | Describe how uncertainty about analytic judgments, inputs, or projections affect findings. Report the effect of choice of discount rate and time horizon, if applicable. | Methods, Page 4-9 |
| **Effect of engagement with patients and others affected by the study** | 25 | Report on any difference patient/service recipient, general public, community, or stakeholder involvement made to the approach or findings of the study | None |
| **Discussion** | | | |
| **Study findings, limitations, generalisability, and current knowledge** | 26 | Report key findings, limitations, ethical or equity considerations not captured, and how these could affect patients, policy, or practice. | Discussion, pages 12-14 |
| **Other relevant information** | | | |
| **Source of funding** | 27 | Describe how the study was funded and any role of the funder in the identification, design, conduct, and reporting of the analysis | Funding, page TBD |
| **Conflicts of interest** | 28 | Report authors conflicts of interest according to journal or International Committee of Medical Journal Editors requirements. | Conflicts of interest/Competing interests, page TBD |

^1^ Husereau D, Drummond M, Augustovski F, et al. Consolidated Health Economic Evaluation Reporting Standards 2022 (CHEERS 2022) Explanation and Elaboration: A Report of the ISPOR CHEERS II Good Practices Task Force. Value Health 2022;25. doi:10.1016/j.jval.2021.10.008
